# Jumping in the human brain: A review on somatic transposition

**DOI:** 10.1016/j.fmre.2025.03.001

**Published:** 2025-03-08

**Authors:** Yufei Zhang, Yanyan Guo, Hangxing Jia, Huijing Ma, Shengjun Tan, Yong E. Zhang

**Affiliations:** a Key Laboratory of Zoological Systematics and Evolution & State Key Laboratory of Animal Biodiversity Conservation and Integrated Pest Management, Institute of Zoology, Chinese Academy of Sciences, Beijing 100101, China; b University of Chinese Academy of Sciences, Beijing 100049, China

**Keywords:** Transposable elements, Somatic transposition, Human brain, Deep learning, Single-cell whole genome sequencing

## Abstract

As a Nobel Prize-winning discovery, transposable elements, or “jumping genes”, have attracted significant interest due to their roles in providing functional coding and regulatory sequences. A longstanding hypothesis suggests that somatic transposition may preferentially occur in the mammalian brain, contributing to neuronal diversity. Here, we aim to provide the latest overview of somatic transposition studies in the human brain. We first introduce the historical context and the limited studies on the functionality of somatic transposition, indicating its pathogenic role. We then highlight the wide variability in somatic transposition rate estimates across studies, discussing the complexities—such as artificial chimeras and the multicopy nature—that contribute to false positive and negative results. We also review the evolving experimental and computational methods designed to mitigate these challenges and briefly cover studies estimating germline transposition rate. Finally, we suggest that advances in single-cell genome amplification methods, coupled with deep learning-based software, could pave the way for more definitive studies on the prevalence and functional role of somatic transposition in the human brain.

## 1 Introduction

Transposable elements (TEs), often termed “jumping genes”, are selfish genetic elements that increase their copy numbers by changing locations within the host genome. Since Dr. Barbara McClintock’s pioneering discovery of TEs in the 1940s [1,2], research interests have been accumulating due to their prevalence in eukaryotic and prokaryotic genomes, their mutagenic properties, and their roles in functional evolution through the provision of coding and regulatory sequences [[3], [4], [5], [6]]. As in many other species, a substantial proportion (∼46%) of the human reference genome is derived from TEs [7]. These TEs, resulting from germline transposition events accumulated over long-term human evolution, may confer domesticated functional roles shaped by natural selection [[8], [9], [10]]. Compared to reference TEs, non-reference TEs, including recently originated and *de novo* germline TE insertions, and somatic TE insertions occurring post-zygotically, are less studied. Unlike germline transposition, somatic transposition is particularly challenging to study as only a subset of cells harbors the corresponding insertions. For humans, three types of TEs can contribute to somatic transposition due to their mobility: autonomous Long interspersed element 1 (L1) and non-autonomous Alu and SINE-VNTR-Alu (SVA), with the transposition of Alu and SVA depending on L1 [11]. In 2005, the Gage lab proposed that somatic transposition may preferentially occur in mammalian neurons, contributing to their functional diversity [12]. Despite extensive efforts over the past two decades, the spatiotemporal mode and functional consequences of somatic transposition remain largely unclear, with limited studies supporting its pathogenic role. To summarize the discoveries in this field, an excellent review was published in 2018, comprehensively covering historical background, studies in various species, functional consequences, and technical challenges [13]. Herein, we provide an updated and focused overview of somatic transposition studies in the human brain, addressing potential functionality, conflicting prevalence estimates, experimental and computational advances, and promising research directions. For comparison, we briefly describe studies estimating germline transposition rates.

## 2 The enigmatic functionality of somatic transposition

Germline and somatic transposition have different functional outcomes. Both types of mutations are subject to natural selection: germline transposition at the organismal level and somatic transposition at the cellular level. Germline TE insertions, especially those long fixed in humans, have undergone extensive positive and negative natural selection. As a result, usually only neutral, nearly neutral, and beneficial insertions have a higher chance to exist. Beneficial insertions become domesticated, providing promoters, enhancers, insulators, and protein sequences. In contrast, somatic TE insertions are subject to selection over a much shorter timescale, potentially resulting in a relatively higher proportion of deleterious or pathogenic insertions.

Consistently, limited studies indicate that somatic transposition tends to be pathogenic. On the one hand, transposition events are often considered deleterious and have been observed in various cancers [14], contributing to both driver and passenger mutations [[15], [16], [17]]. Similarly, increased transposition rates have been noted in neurodevelopmental disorders such as schizophrenia [18] and Rett syndrome [19]. However, neurological disorders may also result from alternative mechanisms, such as the accumulation of TE-derived transcripts or DNA [[20], [21], [22]]. On the other hand, in 10 cases, a single somatic TE insertion has been implicated in directly causing diseases, such as hereditary tumors or immune diseases [[23], [24], [25], [26], [27], [28], [29], [30], [31], [32]].

Nonetheless, some studies suggest a beneficial role for somatic transposition. The most influential one among them is the aforementioned Gage study, which proposed that transposition preferentially occurs during normal brain development, contributing to neuronal diversity [12,[33], [34], [35]]. However, there is *hitherto* no direct evidence supporting the beneficial role of transposition in brain development.

## 3 The uncertain rate of somatic transposition

The functional impact of somatic transposition in the brain remains unclear, partly due to the uncertainty in transposition rates. Extensive efforts have been made to identify somatic transposition events in humans, particularly in the brain (Table 1). Transposition has been detected in various regions, including the cerebral cortex and hippocampus, and across various cell types, such as neurons and glia [36,37]. However, the reported quantities and rates of transposition vary widely, ranging from 0 to over 80,000 per bulk study, and 0.04 to 80 insertions per single cell (Table 1). Even within the same brain region and cell type, discrepancies are notable; for instance, the per-cell rate for neurons in the cerebral cortex in healthy individuals ranges from 0.07 [38] to 16.3 [36]. This large contrast may stem from differences in the study design, *e.*g*.*, sequencing depth (especially across bulk samples), or analytical frameworks (see following sections). Notably, transposition rates have been consistently evaluated for both neurons and glia within the same studies, giving conflicting results. Some studies showed similar rates: 0.1–0.2 insertions per cell in [39], ∼1 insertion per cell in [37], while others showed neurons contained more insertions than glia: 13.7 insertions per cell vs*.* 6.5 insertions per cell in [36].

**Table 1:** **Estimates of human somatic transposition rates**.

Study^a^	Sample age^b^	Sequencing strategy		Computational method	Tissue^c^	Estimated somatic transposition rates^d^			
						L1		Alu	SVA
Coufal et al. [42]	postnatal	bulk	qPCR	–	HIP	80/cell		–	–
Baillie et al. [43]	postnatal	bulk	RC-seq^e^	customized pipeline	HIP, CN	7743/7 samples	0.04/cell^g^ [44]	13,692/7 samples	1350/7 samples
Bundo et al. [18]	postnatal	bulk	WGS	customized pipeline	PFC	2600/3 samples		–	–
					PFC from SZ patients	4213/3 samples		–	–
Kurnosov et al. [45]	postnatal	bulk	targeted sequencing	customized pipeline	CB	1651/1 sample		1317/1 sample	–
					FC	462/1 sample		2138/1 sample	–
					SVZ	1133/1 sample		1308/1 sample	–
					DG	3100/1 sample		2984/1 sample	–
					myocardium	1151/1 sample		1243/1 sample	–
Upton et al. [36]	postnatal	bulk	RC-seq^e^	customized pipeline	liver	175/4 samples		–	–
Jacob-Hirsch et al. [46]	–	bulk	WGS	customized pipeline	HIP, CB, OC	1911/5 samples		–	–
					CB, OC, FC, SEGAs from AT, NSA, Rett, SEGA, and TSC patients	84,495/15 samples		–	–
Muñoz-Lopez et al. [47]	embryonic and newborn	bulk (MDA)^f^	RC-seq^e^, ATLAS-seq^e^	customized pipeline	ICM of blastocysts	1/2 samples^h^		–	–
			RC-seq^e^		placenta	6/10 samples^h^		–	–
Zhao et al. [19]	postnatal	bulk	HAT-seq^e^	customized pipeline	PFC (neurons)	3170/5 samples	1.22/cell^g^	–	–
					PFC (neurons) from Rett patients	3291/5 samples	1.36/cell^g^	–	–
					heart	1170/5 samples	0.54/cell^g^	–	–
					heart from Rett patients	580/2 samples	0.69/cell^g^	–	–
					eye from Rett patients	563/2 samples	0.61/cell^g^	–	–
					fibroblast from Rett patients	411/1 sample	0.69/cell^g^	–	–
Zhu et al. [41]	fetal	bulk	WGS	RetroSom [41]	cortical tissues (neurons)	0/1 sample		0/1 sample	0/1 sample
					cortical tissues (astrocytes)	0/1 sample		0/1 sample	0/1 sample
					heart	0/1 sample		0/1 sample	0/1 sample
	postnatal				STG (neurons)	0/3 samples		0/3 samples	0/3 samples
					STG (glia)	0/3 samples		0/3 samples	0/3 samples
					STG (neurons) from SZ patients	2/2 samples^h^		0/2 samples	0/2 samples
					STG (glia) from SZ patients	0/2 samples		0/2 samples	0/2 samples
					heart	0/1 sample		0/1 sample	0/1 sample
					fibroblast	0/2 samples 0/2 samples		0/2 samples	0/2 samples
					fibroblast from SZ patients			0/2 samples	0/2 samples
Berteli et al. [48]	germline	bulk (MDA)^f^	TIPseq^e^	TIPseqHunter [49]	sperm	17/10 samples^h^		–	–
Möhner et al. [50]	postnatal	bulk	RDA^e^	customized pipeline	PFC	–		–	5149/2 samples
					HIP	–		–	4168/2 samples
					CF	–		–	2614/2 samples
					OB	–		–	6765/2 samples
					CB	–		–	4378/2 samples
Ramirez et al. [51]	postnatal	bulk	WGS (ONT)	TLDR [52]	FC from healthy individuals and AD patients	163/18 samples (L1+Alu+SVA)			
Wallace et al. [53]	newborn	bulk	WGS	MELT [54]	placenta from healthy live births	2/6 samples		0/6 samples	0/6 samples
					placenta from live births with FGR	1/4 samples		0/4 samples	0/4 samples
					placenta from stillbirths with FGR	2/7 samples		0/7 samples	0/7 samples
Evrony et al. [38]	postnatal	single-cell (MDA)	L1-IP^e^	customized pipeline	300 neurons from FC and CN	0.07/cell^h^		–	–
Evrony et al. [40]	postnatal	single-cell (MDA)	WGS	scTea [40]	16 neurons from PFC	0.18/cell^h^ [39]		0/cell	0/cell
Upton et al. [36]	postnatal	single-cell (MALBAC)	RC-seq^e^	customized pipeline	92 neurons from HIP	13.7/cell^h^	0.18/cell^g^ [39]	–	–
					22 glia from HIP	6.5/cell^h^	0.20/cell^g^ [39]	–	–
					35 neurons from FC	16.3/cell^h^	0.25/cell^g^ [39]	–	–
					21 neurons from HIP of AGS patients	8/cell^h^	0.14/cell^g^ [39]	–	–
Erwin et al. [37]	postnatal	single-cell (MDA)	SLAV-seq^e^	customized pipeline	40 neurons from HIP	0.91/cell^h^		–	–
					23 glia from HIP	1.66/cell^h^		–	–
					15 neurons from FC	0.83/cell^h^		–	–
					11 glia from FC	0.78/cell^h^		–	–
Muñoz-Lopez et al. [47]	embryonic and newborn	single-cell (MDA)	RC-seq^e^, ATLAS-seq^e^	customized pipeline	6 cells from ICM of blastocysts	0/cell^h^		–	–
Sanchez-Luque et al. [55]	postnatal	single-cell (MDA)	WGS, RC-seq^e^, L1-IP^e^	TEBreak [56]	24 neurons from HIP	0.04/cell^h^^,^^i^		0/cell	0/cell
Nam et al. [57]	postnatal	clones from single cells	WGS	MELT [54], TraFiC-mem [58], DELLY [59] and xTea [60]	140 HSC clones	0.007/cell^i^		0/cell	0/cell
					341 fibroblast clones	0.04/cell^i^		0.02/cell^i^	0/cell
					406 normal colorectal clones from colorectal cancer patients	3.04/cell^i^		0.005/cell^i^	0/cell

a Studies are first ordered by bulk vs*.* single-cell and then by publication year

b To simplify, we used “postnatal” to refer to children or adult samples

c HIP, hippocampus; CN, caudate nucleus; PFC, prefrontal cortex; CB, cerebellum; FC, frontal cortex; SVZ, subventricular zone; DG, dentate gyrus; OC, occipital cortex; ICM, inner cell mass; STG, superior temporal gyrus; CF, calcarine fissure; OB, olfactory bulb; HSC, hematopoietic stem and progenitor cells; SZ, schizophrenia; AT, ataxia-telangiectasia; NSA, non-syndromic autism; Rett, Rett syndrome; SEGA, subependymal giant cell astrocytoma; TSC, tuberous sclerosis complex; AD, Alzheimer's disease; FGR, fetal growth restriction; AGS, Aicardi-Goutières syndrome

d “-” represents that the sequencing strategy or the computational method could not detect this type of TE

e A targeted sequencing strategy was used

f Multiple displacement amplification (MDA) was used for amplifying DNAs from bulk samples. In addition, MALBAC stands for multiple annealing and looping-based amplification cycles

g Two estimates were available in the original analysis or the reanalysis

h The somatic transposition rate was corrected by PCR validation

i The original work did not provide an estimate, so we calculated it as the total number divided by cell counts.

The high uncertainty of transposition rates means that the temporal mode of somatic transposition events, or whether they biasedly occur in some developmental stage, is also unknown. Actually, as of 2024, only five somatic TE insertions have been timed based on their frequencies and distributions in the brain: one during the embryogenesis (morula) stage [19], three in neuroepithelial cells during initial brain organogenesis [40,41], and one in a neocortical progenitor at a relatively late stage [40]. Despite the small sample size, this dataset suggests that somatic transposition may preferentially occur during early brain development.

Transposition rate studies in somatic tissues other than the brain are scarce (Table 1). However, tissues from the heart, liver, and fibroblasts have been used as controls in brain or cancer studies [14,19]. Only one study estimated transposition rates across the brain and other tissues, finding that the per-cell rate in prefrontal cortex neurons (1.22–1.36) was higher than in heart, eye, and fibroblasts (0.54–0.69) [19]. Whether this high neuronal activity is consistent across studies or in a broader tissue panel requires further investigation.

## 4 The overall framework for somatic TE insertion identification

**Fig. 1:**
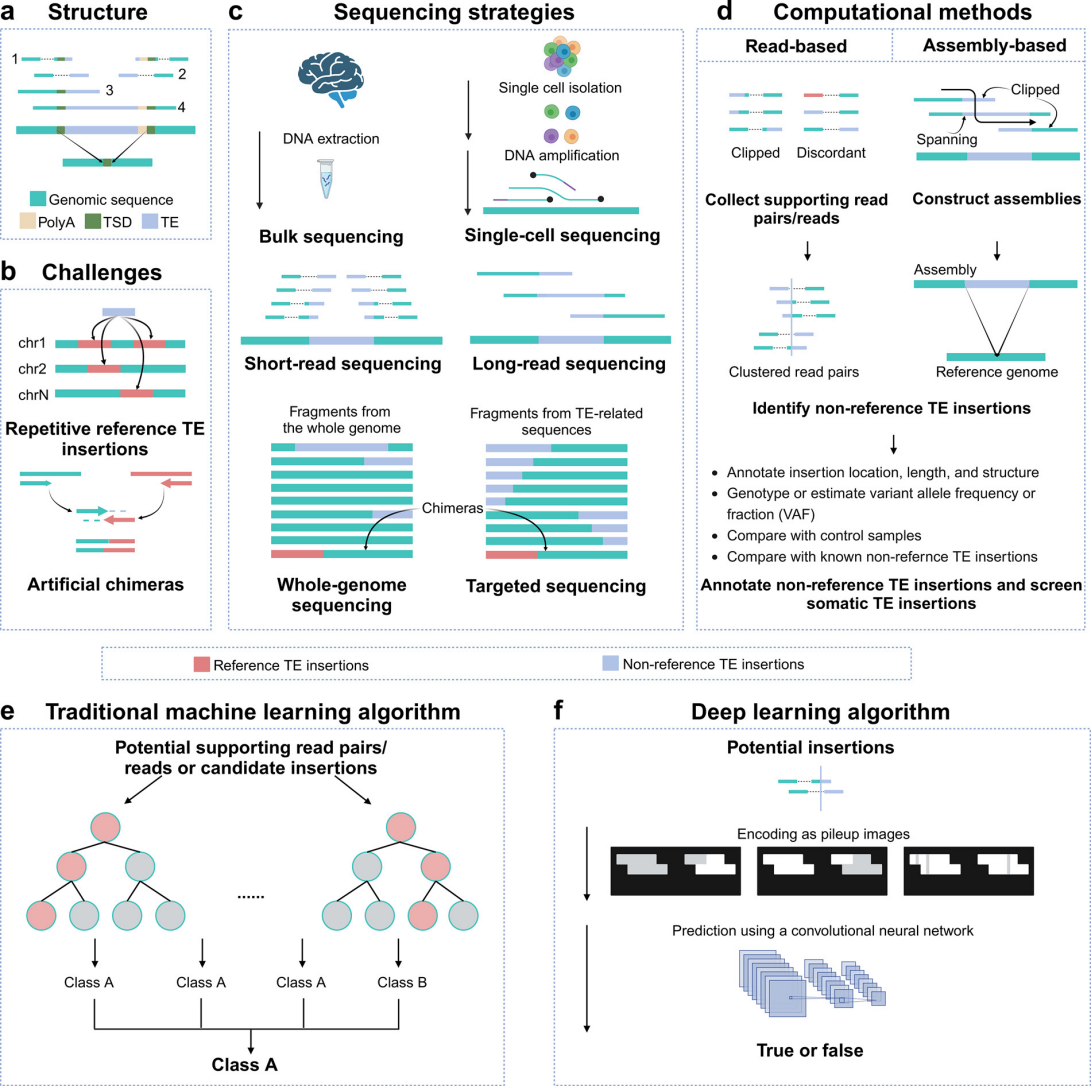
**Identification of somatic TE insertions**. (a) Schematic of a typical non-reference TE insertion. Flanking TSDs, the TE sequence, a polyA tail, and corresponding supporting read pairs/reads are shown. 1: clipped/split read pairs from short-read sequencing; 2: discordant read pairs from short-read sequencing; 3: a clipped/split read from long-read sequencing; 4: a spanning read from long-read sequencing. (b) Two typical challenges in somatic TE insertion analyses: multi-mapping issues caused by the multicopy nature of TEs, and artificial chimeras emerging during DNA amplification and library preparation. (c) Sequencing strategies across three dimensions. (d) Overview of read-based and assembly-based computational methods. Note that read-based methods for long-read sequencing are analogous to those employed for short-read sequencing and are therefore omitted from this figure. (e) A random forest model. The model, integrating predictions from multiple decision trees, is used for the classification of supporting read pairs/reads or candidate insertions. (f) A convolutional neural network (CNN) model. The features of a potential insertion are encoded and sent to a CNN model for classification.

Detecting somatic TE insertions involves identifying sequence features of transposition. For L1, transposition relies on target-primed reverse transcription (TPRT), resulting in target site duplications (TSDs) flanking the insertion site and the incorporation of a polyA tail (Fig. 1a). These hallmark sequence features enable the identification of TE insertions in paired-end short-read sequencing by generating two types of supporting read pairs: clipped/split read pairs and discordant read pairs (Fig. 1a). A clipped read pair includes at least one clipped read, where one segment comes from the genomic sequence and the other from the inserted TE sequence. A discordant read pair comprises two reads with conflicting alignments: one read originates solely from the genomic sequence adjacent to the insertion site, while the other read entirely comes from the TE. Additionally, some clipped short-read pairs can capture both sides of TE insertions, especially for small TEs like Alu or severely truncated L1. In long-read sequencing, supporting reads include clipped/split reads and spanning reads capable of capturing entire TE insertions (Fig. 1a).

Although the framework to identify somatic TE insertions seems straightforward, challenges exist. First, TEs are notoriously difficult to analyze due to their multicopy nature (Fig. 1b). Reads from TE insertions close to preexisting reference TEs are typically unmappable due to alignment ambiguity [61]. Second, artificial chimeras can emerge when two genomic fragments are fused due to template switching during various steps of sequencing library preparation (Fig. 1b), such as PCR in bulk libraries or amplification cycles in single-cell libraries [39]. If one fragment involves a TE, the chimera can mimic signals like clipped/split reads or discordant read pairs. Third, by definition, somatic TE insertions are present in only a subset of cells. In bulk sequencing data from many cells, true positive signals can be hard to identify due to limited supporting reads or read pairs, which may be further obscured by mapping or chimera issues.

## 5 Experimental advancement to identify somatic TE insertions

To address these challenges, numerous efforts have been made to improve experimental methods for detecting somatic transposition. The initial report by the Gage lab detected somatic transposition in rodent brains using an engineered human L1 reporter system (GFP, [12]), which may not reflect *in vivo* transposition in humans. Subsequent work implemented quantitative PCR (qPCR) to quantify somatic transposition rates in human brains [42]. However, this approach could be confounded by the accumulated TE sequences which are not integrated into the genome, leading to an overestimation of the rate [13,62]. Consistently, the highest estimate of 80 insertions per cell was generated by qPCR (Table 1). Therefore, subsequent studies have generally taken sequencing-based approaches to detect supporting reads (*e.*g*.*, clipped/split reads) and identify somatic transposition, evolving along three dimensions (Fig. 1c).

First, compared to bulk sequencing, single-cell sequencing is gaining popularity. A number of studies have used this approach to neurons or glial cells in brain regions such as the hippocampus [36,37,55], cerebral cortex [[36], [37], [38],^40^], and caudate nucleus [38] (Table 1). This approach increases the likelihood of detecting TE insertions shared by a small proportion of cells, especially when a large number of cells are sequenced. Moreover, reads derived from chimeras and TE insertions are often distinguishable, as chimeras typically do not reach the expected variant allele frequency or fraction (VAF) of 0.5—unless they are generated during the early amplification cycles. Single-cell sequencing also comes with tradeoffs, such as high cost and the tendency of whole-genome amplification methods (*e.*g*.*, MDA and MALBAC, Table 1) to under-amplify TE regions which cause false negative calls of TE insertions [63]. Studies on mouse neurons and human colorectal epithelial cells have converted single-cell sequencing to bulk sequencing by sequencing clones developed from single cells to control chimeras or uneven DNA amplification [57,64]. This strategy is generally unsuitable for non-dividing cells including mature adult neurons, but it could be applicable to reprogrammed neurons [64]. Additionally, generating enough clones is labor-intensive, and somatic transposition may occur during clone development.

Second, various targeted sequencing techniques have been developed to enrich fragments containing TE sequences and capture more signals associated with somatic transposition in brain regions like the hippocampus [36,37,43,50,55] and cerebral cortex [[36], [37], [38],^45^,^50^] (Table 1). Techniques like RC-seq [43] use probes to capture fragments from active TE subfamilies and amplify them for sequencing. Other techniques, such as L1-IP [38], SLAV-seq [37], and HAT-seq [19], design specific primers to enrich the insertions via PCR. Although the enrichment substantially lowers costs by removing unrelated sequences, it generally cannot target all active TEs from L1, Alu, or SVA, leading to false negative calls. Thus, whole-genome sequencing approaches are still actively used.

Third, short- and long-read sequencing approaches have been co-developed. Most somatic transposition studies, including those in Table 1, have been performed with short-read sequencing due to its low cost. However, long reads can easily span the whole TE insertion, addressing multi-mapping ambiguity and differentiating chimeras based on the absence of TSDs. Thus, it is well-known that long-read sequencing is more suitable for detecting various structural variations, including TE insertions [[65], [66], [67], [68], [69]]. With the continuous drop in sequencing prices, whole-genome long-read sequencing has recently been employed to identify somatic TE insertions in the human frontal cortex for the first time [51]. To harness the advantages of long reads while controlling costs, several studies have developed targeted long-read sequencing approaches. In one study, specific gRNAs were designed to cleave target TE sequences with Cas9, followed by bulk sequencing, achieving a read length N50 ranging from 14.9 to 32.3 kb, which substantially improved the resolution of insertion structures when identifying germline TE insertions from human cell lines [70]. Another team combined TE enrichment with single-cell long-read sequencing to detect somatic L1 insertions in mouse breast cancer cells [71]. These techniques should also be applicable for detecting somatic transposition in the brain.

Notably, these three dimensions—bulk vs*.* single-cell, whole-genome vs*.* targeted, and short-read vs*.* long-read—can be combined as needed, providing a versatile approach to study transposition in somatic tissues including brain.

## 6 Computational advancement to identify somatic TE insertions

In parallel with the blossoming experimental developments dedicated to somatic transposition, computational methods for identifying non-reference TE insertions are also under active development, although they often do not directly differentiate between germline and somatic transposition. Table 2 summarizes up to 25 methods, developed or optimized in the past 5 years, for identifying non-reference TE insertions. Among them, TEBreak [56], TLDR [52], and RretroSom [41] have been used to detect somatic transposition in the human brain (Table 1), while other brain-related studies employed customized pipelines. These tools or pipelines are certainly also suitable for non-brain tissues. Additionally, targeted sequencing studies capturing only one side of TE insertions limited the use of tools requiring evidence from both sides. Since many tools have been extensively reviewed [60,72,73], we will only focus on their core strategies and the role of machine learning algorithms.

**Table 2:** **Computational methods for identifying non-reference TE insertions in the human genome**.

Tool	Study^a^	Latest update	Type	Read type	Strategy	Genotyping	Notes
Mobster	Thung et al. [81]	2022	TE	Short-read	Read-based	No	–
TEBreak	Carreira et al. [56]	2023	TE	Short-read	Read-based	VAF^b^	–
MELT	Gardner et al. [54]	2020	TE	Short-read	Read-based	Yes	–
RelocaTE2	Chen et al. [82]	2020	TE	Short-read	Read-based	Yes	–
STEAK	Santander et al. [83]	2019	TE	Short-read	Read-based	No	–
AluMine	Puurand et al. [84]	2021	Alu	Short-read	Read-based	Yes	–
ERVcaller	Chen and Li [85]	2024	TE	Short-read	Read-based	Yes	–
MEScanner	Loh et al. [86]	2019	TE	Short-read	Read-based	-c	–
TIP_finder	Orozco-Arias et al. [87]	2021	TE	Short-read	Read-based	No	–
TypeTE	Goubert et al. [88]	2021	TE	Short-read	Read-based	Yes	–
RetroSom	Zhu et al. [41]	2019	TE	Short-read	Read-based	-c	Random forest is used for extracting supporting read pairs.
TEMP2	Yu et al. [89]	2024	TE	Short-read	Read-based	VAF^b^	–
xTea	Chu et al. [60]	2023	TE	Short-read and long-read	Read-based	VAF^b^	Random forest is used for genotyping.
DeepMEI	Xu et al. [80]	2024	TE	Short-read	Read-based	Yes	CNN is used for identifying insertions.
INSurVeyor	Rajaby et al. [90]	2024	SV^d^	Short-read	Read-based	Yes	–
nanomonsv	Shiraishi et al. [91]	2024	SV^d^	Short-read and Long-read	Read-based	No	–
Total ReCall	Solovyov et al. [92]	-e	TE	Short-read	Read-based	-e	–
McClintock 2	Chen et al. [93]	2024	TE	Short-read	Read-based	VAF^b^	McClintock is a meta-pipeline integrating 12 tools.
rMETL	Jiang et al. [94]	2024	TE	Long-read	Read-based	Yes	–
PALMER	Zhou et al. [95]	2023	TE	Long-read	Read-based	No	–
TLDR	Ewing et al. [52]	2023	TE	Long-read	Read-based	No	–
MEIGA-PAV	Ebert et al. [96]	2022	TE	Long-read	-f	No	–
ricME	Ma et al. [97]	2023	TE	Long-read	Read-based	-c	–
somrit	D'Costa and Simpson [98]	2023	TE	Long-read	Read-based	No	–
GraffiTE	Groza et al. [99]	2024	TE	Long-read	Read-based and assembly-based	VAF^b^	–

a Studies are first ordered by short- vs*.* long-read and then by publication year

b VAF is also given when genotyping

c The paper does not explicitly state whether the method can perform genotyping

d This tool could identify various SVs including TE insertions

e The source code has not been released

f MEIGA-PAV relies on several upstream tools.

Tools designed for short-read sequencing data generally rely on the aforementioned clipped and discordant read pairs (Fig. 1d). These supporting read pairs are extracted based on the read-to-genome alignments, followed by realignment to consensus sequences of active TE subfamilies. Properly aligned reads are extracted and clustered by insertion sites as candidate non-reference TE insertions. Two strategies have been developed to further identify somatic transposition among non-reference TE insertions. First, a few tools such as xTea offer a somatic mode that processes both experimental and control bulk samples to retain insertions not shared as candidate somatic TE insertions [60]. If the somatic mode is unavailable, both samples can be analyzed separately, and shared insertions can be manually removed. This strategy could be extended to single-cell sequencing data, with insertions specific to a proportion of cells identified as somatic TE insertions. Second, for bulk whole-genome sequencing data, TE insertions with a VAF significantly lower than 0.5 (expected for heterozygous germline mutations) are deemed candidate somatic TE insertions. Notably, for both strategies, the somatic TE calls can be examined for overlaps with databases collecting non-reference germline TE insertions to exclude polymorphic germline insertions (Fig. 1d, [54,[74], [75], [76], [77]]).

Tools designed for long-read sequencing data rely on either reads or assemblies. Read-based tools are similar in design to those for short-read sequencing data. Assembly-based tools compare assemblies generated from sequencing data with the reference genome to identify TE insertions (Fig. 1d). However, these methods often cannot detect low-frequency somatic TE insertions in bulk data, as these insertions are less likely to be assembled.

The application of machine learning, especially deep learning techniques, has shown superior performance in single nucleotide variant (SNV) and structural variation (SV) detection, as evidenced by tools like DeepVariant [78] and SVision-pro [79]. Similarly, despite limited studies applying machine learning to TE insertion detection, its efficacy in supporting read pair identification, genotyping, and insertion detection is evident. Only three tools have directly applied machine learning to detect TE insertions in the human genome, utilizing random forests and convolutional neural network (CNN) models (Table 2). Specifically, RetroSom employed random forests to extract supporting read pairs, generating a collection of decision trees based on features such as sequence alignment to TE consensus sequences, thereby reducing false positives (Fig. 1e, [41]). Similarly, xTea utilized random forests for insertion genotyping, achieving 99.7% accuracy in testing data (Fig. 1e, [60]). Since CNNs are ideal for image-like data, DeepMEI encoded nucleotide bases, base quality, and mapping quality as pileup images to detect TE insertions (Fig. 1f, [80]).

The development of these computational methods largely reflects advances in experimental techniques. For instance, the emergence of long-read sequencing datasets [51] has driven the need for specialized computational tools (Table 2).

## 7 The rate of germline transposition

*De novo* germline transposition, occurring across generations, has been extensively studied before somatic transposition and is recognized for its pathogenic potential [100]. In humans, germline transposition rates have been estimated using either conventional evolutionary methods or the recently developed trio-sequencing approach (Table 3). Evolutionary methods rely on parameters such as the mutation rate, transposition proportion, neutral molecular clock, and evolutionary time [[101], [102], [103]]. In contrast, trio-sequencing is more straightforward by directly identifying mutations present only in offspring. However, this method has two limitations: (1) trio cohorts often come from disease pedigrees, introducing potential sampling bias; and (2) early somatic transposition events at high frequency may be misclassified as germline insertions [104].

**Table 3:** **Estimates of human*****de novo*****germline transposition rates**.

Study	Estimation strategy^a^	Computational method^b^	Estimated germline transposition rates (insertion/birth)^c^		
			L1	Alu	SVA
Deininger et al. [105,106]	evolutionary methods	–	-	1/100	–
Kazazian [102]	evolutionary methods	–	1/100–1/8		
Li et al. [107]	evolutionary methods	–	1/28–1/2.4		
Brouha et al. [108]	transposition activity assay analysis	–	1/33–1/2	–	–
Cordaux et al. [101]	evolutionary methods	–	–	1/20	–
Xing et al. [109]	evolutionary methods	–	1/212	1/21	1/916
Ewing et al. [103]	evolutionary methods	–	1/270- 1/95	–	–
Huang et al. [110]	evolutionary methods	–	1/108	–	–
Feusier et al. [111]	trio data analysis of healthy individuals	MELT [54], RUFUS [112], and TranSurVeyor [113]	1/63	1/40	1/63
Gardner et al. [114]	evolutionary methods and trio data analysis of patients with developmental disorders	MELT [54]	1/14–1/12		
Belyeu et al. [115]	trio data analysis of healthy individuals and ASD patients	Lumpy [116], Manta [117], Delly [59], Whamg [118], MELT [54], GATK-SV [76,119]	1/231	1/42	1/309
Borges-Monroy et al. [30]	trio data analysis of healthy individuals and ASD patients	xTea [60]	1/117	1/29	1/206
Niu et al. [120]	evolutionary methods	MELT [54]	1/17–1/16		
Chu et al. [104]	trio data analysis of birth defect and childhood cancer patients	xTea [60]	1/108	1/34	1/93

a ASD, autism spectrum disorder

b “-” represents that the estimation did not utilize computational methods for sequencing data

c “-” represents that the estimation did not include this type of TE.

Studies on germline transposition rates differ from those on somatic transposition in two key ways (Tables 1, 3). First, while somatic transposition rate estimates vary by several orders of magnitude (Table 1), germline rates show less variation, likely because their high frequency makes detection easier, or their experimental and computational frameworks are more consistent with each other. Both evolutionary and trio-sequencing methods estimate the total germline transposition rate of L1, Alu, and SVA at roughly one event per tens of births (Table 3). Second, Alu transposes more frequently than L1 in the germline (Table 3), consistent with its high genomic copy number, whereas L1 shows higher transposition rates in most somatic studies (Table 1). This discrepancy may reflect distinct regulatory mechanisms between germline and somatic tissues or experimental/computational differences, warranting further investigation.

## 8 Conclusion and future perspectives

As early as 2005, the Gage group implemented the L1-GFP reporter system and first hypothesized preferential transposition in the brain and its potential benefits [12]. Over the following two decades, advances in experimental and computational methods have enhanced the understanding of somatic transposition in three ways (Table 1). First, short-read sequencing reproduced TE insertions in the normal human brain [43] and confirmed earlier correlations between somatic transposition and neurological disorders (*e.*g*.*, L1-GFP or PCR [19,121]). Second, single-cell short-read sequencing provided a relatively more accurate transposition rate estimate, much lower than the initial 80 insertions per cell (Table 1). The previously mentioned hypothesis was also moderated: while brain transposition rates may be low, insertions in a few neurons could still substantially impact function due to neuronal circuitry [22]. Third, single-cell short-read sequencing enabled high-resolution temporal analysis, showing that three out of five timed insertions occurred during early brain organogenesis [19,40,41]. Additionally, the recent application of long-read sequencing has resolved somatic TE insertion sequences in the human brain [51]. Both temporal and full-sequence data facilitate interpreting functional impacts. Despite these three lines of progress, the precise rate and function of transposition remain unclear. Discrepancies in reported rates arise from sample heterogeneity (Table 1) and differences in experimental and computational methods. Based on previous work, we identify two promising directions for future exploration.

On the experimental front, further development of low-bias, low template-switching single-cell whole-genome amplification methods is crucial. As mentioned earlier, somatic transposition events present in a subset of cells are difficult to detect in bulk sequencing data, making single-cell sequencing the preferred approach. However, amplification methods used in previous single-cell studies, such as MDA and MALBAC, are associated with underrepresented TE regions and template-switching chimeras [39,63]. Compared to these methods, Linear Amplification via Transposon Insertion (LIANTI) performs relatively better [122]. However, this method is intrinsically complex, involving whole-genome transcription and reverse transcription, and has not been used in somatic transposition studies. In principle, LIANTI could be streamlined, as often shown in the development of sequencing library methods (*e.*g*.*, [68]). A simplified version of LIANTI or other new low-bias, low-switching methods would enable the generation of high-quality single-cell whole-genome data.

On the computational side, a wave of deep learning-based methods dedicated to somatic transposition detection is on the horizon. Many previous methods have essentially wrapped up sequence alignment with empirical rules without incorporating machine learning algorithms. As shown by the rapid progress in structural variation detection software development, deep learning or neural network-based approaches offer superior performance [79,123]. Since tools specifically designed for TE insertions generally outperform general structural variation detection tools, deep learning-based TE insertion detectors like DeepMEI [80] warrant further development, especially in the following four directions. First, current tools apply machine learning to only one step of the identification process. New tools could benefit from integrating machine learning across multiple steps. Second, the lack of standardized training and benchmark datasets hinders effective model training and evaluation. An ideal dataset would include sufficient artificial chimeras to enhance performance in distinguishing true signals from overwhelming false positives. Third, challenges in TE alignment necessitate the development of novel aligners, more complete reference genomes, and improved TE consensus sequences. Fourth, end-to-end models [124], which directly identify TE insertions from raw sequencing data and minimize preprocessing and reliance on sequence alignment, offer great potential.

With these evolving experimental and computational methodologies and the rapidly declining costs of short- and long-read sequencing, the landscape of somatic transposition in the brain and other human organs is likely to be revealed soon. Precise detection of transposition will pave the way for subsequent functional studies. Additionally, the study of somatic mutations, especially SNVs, has already become an active field, often referred to as somatic mosaicism. Significant insights have been gained by studying somatic SNVs, such as the early cellular division asymmetry of the brain [125] or the rescue effect of somatic mutations for preexisting germline mutations [126]. Novel insights are expected from an in-depth exploration of somatic transposition. One particularly relevant question is whether transposition could sometimes be beneficial, as hypothesized two decades ago [12]. By studying both small mutations like SNVs and large mutations like TE insertions, we can gain a more complete understanding of how development occurs despite inevitable mutational perturbations.

## Declaration of competing interest

The authors declare that they have no conflicts of interest in this work.
